# Elevated Sporulation Efficiency in Fission Yeast *Schizosaccharomyces japonicus* Strains Isolated from *Drosophila*

**DOI:** 10.3390/jof7050350

**Published:** 2021-04-29

**Authors:** Taisuke Seike, Natsue Sakata, Fumio Matsuda, Chikara Furusawa

**Affiliations:** 1 Center for Biosystems Dynamics Research, RIKEN, 6-2-3 Furuedai, Suita, Osaka 565-0874, Japan; natsue.sakata@gmail.com (N.S.); chikara.furusawa@riken.jp (C.F.); 2 Department of Bioinformatic Engineering, Graduate School of Information Science and Technology, Osaka University, 1-5 Yamadaoka, Suita, Osaka 565-0871, Japan; fmatsuda@ist.osaka-u.ac.jp; 3 Universal Biology Institute, The University of Tokyo, 7-3-1 Hongo, Bunkyo-ku, Tokyo 113-0033, Japan

**Keywords:** fission yeast, *Schizosaccharomyces japonicus*, mating, sporulation, pheromone, *Drosophila*

## Abstract

The fission yeast *Schizosaccharomyces japonicus*, comprising *S. japonicus* var. *japonicus* and *S. japonicus* var. *versatilis* varieties, has unique characteristics such as striking hyphal growth not seen in other *Schizosaccharomyces* species; however, information on its diversity and evolution, in particular mating and sporulation, remains limited. Here we compared the growth and mating phenotypes of 17 wild strains of *S. japonicus*, including eight *S. japonicus* var. *japonicus* strains newly isolated from an insect (*Drosophila*). Unlike existing wild strains isolated from fruits/plants, the strains isolated from *Drosophila* sporulated at high frequency even under nitrogen-abundant conditions. In addition, one of the strains from *Drosophila* was stained by iodine vapor, although the type strain of *S. japonicus* var. *japonicus* is not stained. Sequence analysis further showed that the nucleotide and amino acid sequences of pheromone-related genes have diversified among the eight strains from *Drosophila*, suggesting crossing between *S. japonicus* cells of different genetic backgrounds occurs frequently in this insect. Much of yeast ecology remains unclear, but our findings suggest that insects such as *Drosophila* might be a good niche for mating and sporulation, and will provide a basis for the understanding of sporulation mechanisms via signal transduction, as well as the ecology and evolution of yeast.

## 1. Introduction

The fission yeast *Schizosaccharomyces japonicus*, which was first isolated from strawberries at a farm of Kyusyu University in Japan [1], is a close relative of the well-studied model organism *Schizosaccharomyces pombe* [2]. However, *S. japonicus* differs from other fission yeast species with respect to several features. First, the size of *S. japonicus* haploid cells is bigger, facilitating observation of chromosome behavior such as condensed mitotic chromosomes [3,4,5]. Second, similar to many fungal species, *S. japonicus* is dimorphic and can exist in the form of both yeast and filamentous (hyphae) cells [6]. Fungal dimorphism is usually associated with pathogenic activity towards animals and plants [7] because a hyphae cell can penetrate into a host cell; however, *S. japonicus* is nonpathogenic to humans. Hyphal growth of *S. japonicus* is triggered by environmental stresses as nutritional starvation [8,9,10] and DNA damage [11]. This response is thought to enable hyphae cells of *S. japonicus* to escape more quickly from harmful stimuli. *S. japonicus* also has unique physiological features such as semi-open mitosis [4], light response [12], and growth via fermentation under anaerobic conditions [13]. Despite these interesting properties, relatively few studies on *S. japonicus* have been reported to date.

More than a decade since *S. japonicus* was isolated by Yukawa and Maki, a variant termed *S. japonicus* var. *versatilis* was isolated from home-canned grape juice in the United States [14]. *S. japonicus* var. *japonicus* and *S. japonicus* var. *versatilis* show some differences in characteristics and phenotypes; for example, sporulated colonies of the former are not stained by iodine vapor [15], whereas *S. japonicus* var. *versatilis* sporulated colonies are stained by iodine vapor [16]. Nevertheless, *S. japonicus* var. *japonicus* and *S. japonicus* var. *versatilis* have been demonstrated to be genetically compatible and can mate with each other following sporulation [17]. Notably, sporulated cells of the cross exhibit an iodine-negative phenotype, suggesting that the negative iodine-staining genotype of *S. japonicus* var. *japonicus* is dominant [17]. The difference in iodine staining phenotype has been attributed to variations in at least two unlinked genes between the two strains, but the mechanism remains unclear [17].

In recent years, genetic methods for studying *S. japonicus* (e.g., transformation [18,19] and spore dissection [20]) and bioresources (e.g., heterothallic strains [15]) have become available. In addition, whole-genome sequences of *S. japonicus* were published by the Broad Institute in 2011 [21]. *S. japonicus* is gradually becoming a popular experimental microorganism in addition to *S. pombe*. However, because only a few *S. japonicus* wild strains have been isolated, mainly from fruits and plants such as grapes and from slime fluxes, information on its diversity and evolution, especially mating and sporulation, remains limited.

Although key aspects of yeast ecology remain poorly elucidated, it has been proposed that insects allow yeasts to disperse [22]. Many yeasts and insects depend similarly on a source of sugar, such as rotting fruit or floral nectar; thus, insects such as fruit flies, which are known to accumulate yeast and bacteria in their crop, may provide a means for immobile yeasts to access sugar sources and transfer between sources [23]. Furthermore, insects may also increase outbreeding between different yeast strains: experimental data have suggested that the gut environment of insects, including fruit flies, might facilitate outbreeding [24,25]. This might also assist in adaptive outbreeding among diverse yeasts in the wild.

In this study, we successfully isolated, for the first time to our knowledge, eight *S. japonicus* var. *japonicus* strains possessing different genetic backgrounds from *Drosophila* species sampled at two distinct locations in Japan. Interestingly, most of the isolated strains were found to have a high-temperature tolerance and to sporulate at high frequency on nitrogen-abundant medium, although the type strain of *S. japonicus* var. *japonicus* does not generally mate under this condition. Sequence analysis subsequently revealed that pheromone-related genes were diversified among the eight strains. Further genetic characterization is needed to understand the mating responses of *S. japonicus* under various culture conditions, but we believe that the information and resources presented in this study will facilitate future studies on signal transduction and the regulation of sporulation, as well as unraveling yeast ecology and evolution in nature.

## 2. Materials and Methods

### 2.1. Yeast Strains, Media, and Culture Conditions

The wild strains of *S. japonicus* examined used in this study are listed in Table 1. Three strains (FY16936, FY32181*, and FY33672) were provided by the National Bio-Resource Project (NBRP), Japan (https://yeast.nig.ac.jp/yeast/top.xhtml (accessed on 16 February 2021)). Six strains (NBRC1646, NBRC1712, NBRC1713, NBRC10527*, NBRC10528*, and NBRC10529*) were obtained from the Biological Resource Center, NITE (NBRC), Japan (https://www.nite.go.jp/en/index.html (accessed on 16 February 2021)). The other strains were isolated by Taisuke Seike and Natsue Sakata from *Drosophila* captured inside RIKEN Center for Biosystems Dynamics Research or Suita Campus at Osaka University, Japan. Strain TS16 (*h^−^ sxa2::kanMX6*) constructed in this study was a derivative of NIG5872 (*h^−^*) [15].

Cells were typically grown in YE medium (5 g/L of Bacto Yeast Extract [BD Bioscience, Sparks, MD, USA] and 30 g/L of D-glucose [Wako Pure Chemicals, Osaka, Japan], supplemented with adenine sulfate [200 mg/L], uracil [200 mg/L], and leucine [100 mg/L]). For the solid medium, 15 g/L of agar [Wako Pure Chemicals] was added to the YE medium to form the YEA medium. Where appropriate, geneticin (G418) disulfate (Nacalai Tesque, Kyoto, Japan) was added to YEA medium at a final concentration of 100 μg/mL. For hyphal induction, YMoA medium (35 g/L of Difco^TM^ yeast morphology agar [BD Bioscience]) was used. For mating and sporulation, the MEA medium (30 g/L of extract malt [Nacalai Tesque] and 15 g/L of agar, pH 5.5) and Edinburgh minimal medium without nitrogen (EMM2−N; 26.77 g/L of EMM−Nitrogen [Sunrise Science Products, Knoxville, TN, USA]) were used. For solid medium, 20 g/L of agar was added to EMM2−N medium to form EMM2−N agar medium. For vegetative growth, hyphal growth, mating, and sporulation, cells were incubated at 30 °C, unless stated otherwise.

### 2.2. Isolation of S. japonicus from Drosophila

To collect large numbers of *Drosophila*, we used commercially available bananas. We placed a piece of ripe bananas in a milk carton and then hung it with a rope on tree branches located approximately 2 m from the ground. After a few days, we covered the milk carton with an aseptic plastic bag and captured *Drosophila* that escaped toward the bag by tapping the carton. Next, in the laboratory, we transferred the obtained *Drosophila* (about ten) in a clean 1.5-mL tube, and then washed their body surface with sterilized water. We homogenized *Drosophila* to obtain samples, including their crops, using a homogenizer pestle (INA•OPTIKA, Osaka, Japan). The samples were diluted with sterilized water and spread onto YEA plates containing 100 µg/mL ampicillin and 100 µg/mL chloramphenicol (to prevent the growth of any bacteria) at 30 °C for 2 days. The colonies that appeared on YEA plates were classified based on colony color (white, yellowish, and red) and surface (diaphanous or opaque), and their morphology was inspected using a microscope. Consequently, we collected several wild yeasts. Most of the isolated strains were budding-type yeasts, such as those from the genera *Candida*, *Pichia*, and *Hanseniaspora*. However, some strains were judged to be the fission yeast *S. japonicus* based on their fission proliferation pattern, larger cell size, and formation of eight spores.

### 2.3. Sequence Analysis of S. japonicus Wild Strains

Genomic DNA was extracted from cultures grown overnight in YE medium by using a Wizard Genomic DNA Purification Kit (Promega, Madison, WI, USA). The D1/D2 divergent domains of the large subunit ribosomal DNA (rDNA) were determined by sequencing using the primers oTS1250/oTS1251 [26] and were used to identify the varieties of *S. japonicus* (Table 1). Each of the DNA fragments containing *map2* (P-factor gene; SJAG_00781), *map3* (M-factor receptor gene; SJAG_01345), and *mam2* (P-factor receptor gene; SJAG_01928) was amplified by using the respective primer sets: oTS1254/1255, oTS1258/1259, and oTS1262/1263 (all primers are listed in Table S1). The PCR products were sequenced by using internally specific primers: oTS1256 (*map2*), oTS1260/oTS1261 (*mam2*), and oTS1264/oTS1265 (*map3*). The sequences obtained were compared with those of the FY16936 strain; the differences are listed in Table 2 and Table S2.

### 2.4. Growth Assay at Various Temperatures

Cells were pre-cultured on YEA medium at 30 °C overnight, harvested in sterilized water, and inoculated at an optical density at 600 nm of 0.1 (OD_600_ = 0.1) into 1 mL of YE medium. Next, 200-µL of cell culture was added to each well of 96-well plates (CELLSTAR 96-Well Plates Polystyrene flat bottom well plates; Greiner Bio-One, Kremsmünster, Austria). During 24 hours of cultivation with continuous shaking in orbital mode at 355 cpm (4 mm) at various temperatures (30, 37, 40, and 42 °C), the OD_600_ of each well containing *S. japonicus* cells was measured every 30 min automatically by using a microplate reader (Synergy^TM^ HTX Multi Mode Microplate Reader; BioTek Instruments, Winooski, VT, USA). The growth assay was performed in quadruplicate for each sample (n = 4), and the mean ± standard deviation (SD) of growth rate (per hour) was calculated as described previously [27].

### 2.5. Spotting Assay

Cells were pre-cultured on YEA medium at 30 °C overnight and then diluted to an OD_600_ of 10. The diluted cultures were added to sterile 96-well plates and four fivefold serial dilutions (OD_600_ of 2.0, 0.4, 0.08, and 0.016) were prepared by adding sterilized water. A 6-µL aliquot of the resulting cell suspensions was spotted onto YEA plates and incubated at 42 °C for 3 days. After incubation, each plate was photographed.

### 2.6. Hyphal Formation

Cells were pre-cultured on YEA medium at 30 °C overnight, harvested in sterilized water, and diluted to an OD_600_ of 0.1. A 5-µL aliquot of the diluted culture was spotted in the center of a YMoA plate, and incubated at 30 °C for 10 days, as described previously [9]. Colonies with hyphal zones were photographed.

### 2.7. Mating Frequency

Cells grown on YEA plates overnight were resuspended in sterilized water at an OD_600_ of 5.0. A 30-μL aliquot of the resulting suspension was spotted onto EMM2−N or YEA plates (including a nitrogen source) and incubated for 48 hours at 30 °C as described previously [20]. The number of cells was counted under a differential interference contrast (DIC) microscope (BX53; Olympus, Tokyo, Japan). Cell types were classified into four groups: vegetative cells (V), zygotes (Z), asci (A), and spores (S). Mating was calculated by using the following equation [28]:

Mating (%) = 100 × (2Z + 2A + S/4)/(V + 2Z + 2A + S/4)

In all cases, *S. japonicus* cells were counted and classified in nine randomly photographed digital images of each strain (>400 cells counted in total), and the mean ± SD was calculated.

### 2.8. Iodine Staining

Cells grown on YEA plates overnight were resuspended in sterilized water at an OD_600_ of 0.1. A 6-μl aliquot of the resulting suspension was spotted onto MEA plates and then incubated at 30 °C for 2 days. The colonies of *S. japonicus* strains on MEA plates were stained by iodine vapor (Wako Pure Chemicals) for 1 min. The stained colonies were photographed.

### 2.9. Plasmid Construction and Transformation

To delete the *sxa2^+^* gene, encoding P-factor-degrading enzyme (SJAG_00975), from *S. japonicus* cells, the 5’-upstream sequence (500 bp) of *sxa2^+^* was first amplified from FY16936 genomic DNA by using the primers oTS9/oTS10 (Table S1), and the BamHI/PacI fragment was cloned into pFA6a-kanMX6 to create pTS2 [pFA6a-kanMX6(Sjsxa2UP)]. Next, the 3’-downstream sequence (1 kb) of the *sxa2^+^* gene was amplified from FY16936 genomic DNA by using the primers oTS61/oTS62, and the DNA fragment was fused to a linearized vector derived from pTS2, which was prepared by inverse PCR using KOD FX Neo (TOYOBO, Osaka, Japan) with primers oTS37/oTS38 by Gibson Assembly (New England Biolabs, Ipswich, MA, USA). The resultant plasmid was called pTS7 [pFA6a-kanMX6(Sisxa2UPDOWN)]. A 3-kb fragment was amplified from pTS7 and purified by using the QIAquick PCR Purification Kit (Qiagen, Tokyo, Japan). The DNA fragment was transformed in a heterothallic M-strain, NIG5872, by electroporation as described previously [18], and transformants were selected on YEA containing G418. Primers oTS9/oTS38 were used to confirm that the ORF of *sxa2^+^* had been deleted.

### 2.10. Shmoo Formation by Pheromone Treatment

P-factor peptides were chemically synthesized (Eurofins Genomics, Tokyo, Japan) [29]. The purity of the preparations was confirmed to be more than 95% by HPLC. Each P-factor was dissolved in dimethyl sulfoxide (DMSO) at a concentration of 500 µM. For the assay, a heterothallic haploid *S. japonicus* strain lacking the endogenous *sxa2^+^* gene was used. The cells were grown in YE medium overnight, washed with sterilized water three times, and then resuspended in EMM2−N medium at OD_600_ of 2.0. The cells were incubated with each P-factor peptide (5 µM) for exactly 24 hours with gentle shaking and then observed by DIC microscopy.

### 2.11. Data Availability

All relevant data are included in the paper and the Supporting Information file (Table S3).

## 3. Results

### 3.1. Isolation of the Fission Yeast S. japonicus var. japonicus from Drosophila

To investigate the diversity of the fission yeast *S. japonicus* in sexual reproduction, we obtained the following wild strains from NBRP and NBRC: for *S. japonicus* var. *japonicus*, the type strain FY16936 (also known as ATCC10660/IFO1609) and four other strains; for *S. japonicus* var. *versatilis*, the type strain FY32181* (also known as ATTC9987/IFO1607) and three other strains (Table 1). These nine strains were isolated mainly from fruits and plants. We, therefore, attempted to isolate additional *S. japonicus* species from an insect source. Because fruit flies feed on immobile yeast and are known to accumulate yeast and bacteria in their crop [1], we attempted to isolate novel *S. japonicus* strains from *Drosophila*. By trapping hundreds of flies with a banana, we successfully isolated eight *S. japonicus* strains from two locations: the campus of RIKEN and Osaka University in Japan (Table 1). We determined the sequences of the D1/D2 domains of rDNA genes in these strains, which confirmed that all of the strains isolated were *S. japonicus* var. *japonicus*. Below, we analyzed similarities and differences in various features of the 17 wild *S. japonicus* strains (Table 1).

### 3.2. Heat Tolerance in Most of the Strains Isolated from Drosophila

**Figure 1:**
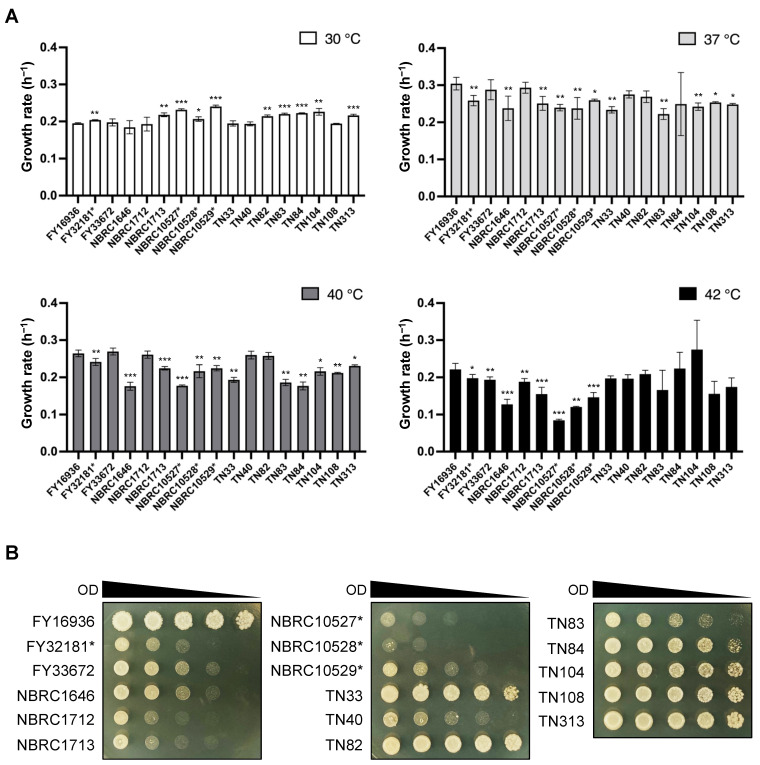
Effects of high temperature on growth. (**A**) Growth rate per hour (h^−1^) of 17 *S. japonicus* strains incubated in YEL medium at various temperatures (30 °C, 37 °C, 40 °C, and 42 °C). Data are the mean ± SD of quadruplicate samples. *t*-test: * *p* < 0.05; ** *p* < 0.01; *** *p* < 0.001. (**B**) Heat tolerance of the strains. Samples were withdrawn from cultures, and the OD_600_ was adjusted to 10. Fivefold serial dilutions (from 5^−1^ to 5^−4^) were prepared in sterilized water, and 6-μL of each sample (the culture at OD_600_=10 and its dilutions) spotted onto YEA plates. The plates were incubated at 42 °C for 3 days and photographed.

In the genus *Schizosaccharomyces*, *S. japonicus* is the only species that can grow at high temperatures (e.g., 40 °C) [13]. We, therefore, compared the growth rate of the 17 *S. japonicus* wild strains under high-temperature conditions. Cells were incubated in YEL medium at 30, 37, 40, and 42 °C, and the OD_600_ was measured with a photo recorder. At 30 °C, *S. japonicus* var. *versatilis* strains grew slightly faster than *S. japonicus* var. *japonicus* strains (e.g., the growth rate of NBRC10529* vs FY16936 was 0.241 ± 0.004 h^−1^ vs 0.195 ± 0.002 h^−1^, respectively); by contrast, most *S. japonicus* var. *versatilis* strains showed slower growth at 42 °C (Figure 1A). Almost all strains of *S. japonicus* grew fastest at 37 °C, consistent with findings from a previous study [6].

To further evaluate tolerance to high temperature, we carried out spot assays of *S. japonicus* cells on YEA medium at 42 °C. These assays indicated that the growth of *S. japonicus* var. *versatilis* strains was severely inhibited at 42 °C (Figure 1B). Better growth was observed for the *S. japonicus* var. *japonicus* strains TN33, TN82–TN84, TN104, TN108, and TN313, as well as FY16936 (Figure 1B). Thus, most of the strains that were isolated from *Drosophila* showed tolerance to high-temperature stress.

### 3.3. Differences in Hyphal Formation among S. japonicus Strains

**Figure 2:**
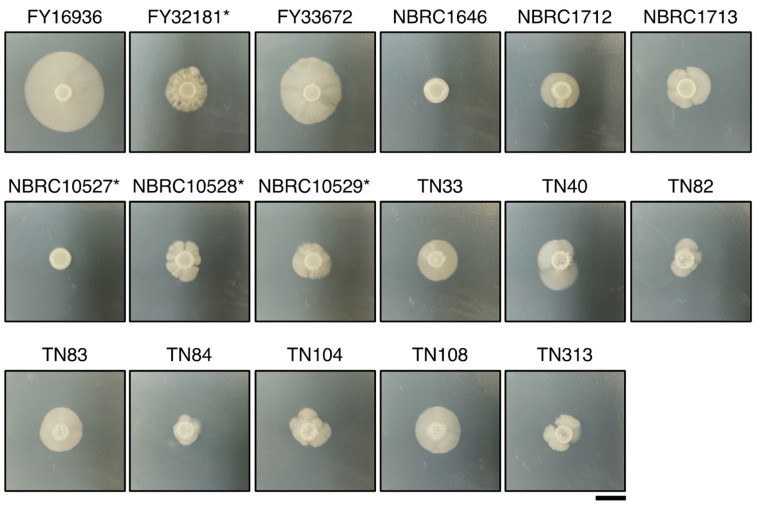
Differences in hyphal formation. Hyphal growth of 17 *S. japonicus* strains incubated on YMoA medium at 30 °C for 10 days. Two strains, NBRC1646 and NBRC10527*, did not form hypha under these conditions. *S. japonicus* var. *versatilis* strains were marked with an asterisk (*). Scale bar, 1 cm.

Unlike other fission yeast species, *S. japonicus* is a dimorphic yeast that can transit from unicellular yeast to filamentous hyphae [6]. Therefore, we examined whether the *S. japonicus* strains could undergo hyphal transition dependent on the culture medium. A conventional medium, yeast morphology agar (YMoA), has been reported to induce a transition to hyphal cells in *S. japonicus* due to nutritional deficiencies [9]; therefore, we spotted *S. japonicus* cells on YMoA and incubated them at 30 °C for 10 days. Among the 17 strains, FY16936, the type strain of *S. japonicus* var. *japonicus*, showed the biggest hyphal zone on the plate, and FY33672 also showed strong induction of hyphae (Figure 2). By contrast, NBRC1646 and NBRC10527* did not show any hyphal zones under this condition (Figure 2). Among the eight strains isolated from flies, the size of the hyphal zone differed to some extent (Figure 2), implying that *S. japonicus* var. *japonicus* strains are diversified in *Drosophila*.

### 3.4. Elevated Sporulation Efficiency in S. japonicus Strains Isolated from Drosophila

**Figure 3:**
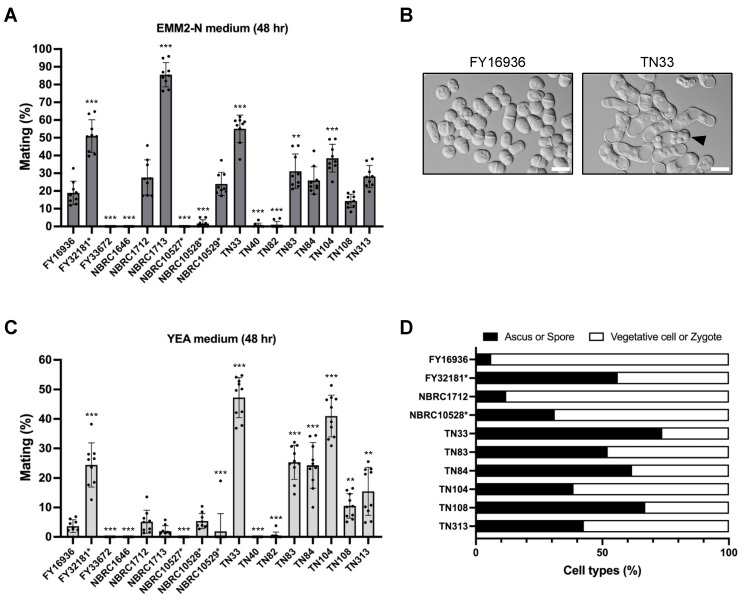
Mating response on different media. Mating frequency of 17 *S. japonicus* strains incubated on EMM2−N (**A**) or YEA (**C**) medium at 30 °C for 48 hours. At least 400 cells were examined for each strain. Data are the mean ± SD of nine samples. (**B**) Micrographs showing FY16936 (wild strain) and TN33 (a strain isolated from flies) incubated on YEA medium at 30 °C for 1 day. Scale bar, 10 µm. (**D**) Cell type proportion of some strains. Black bars indicate ascus or spore; white bars indicate vegetative cell or zygote. *t*-test: * *p* < 0.05; ** *p* < 0.01; *** *p* < 0.001.

Next, we compared the mating efficiency of the 17 *S. japonicus* wild strains on a standard sporulation medium, EMM2−N. After 48 hours of incubation at 30 °C, some *S. japonicus* strains mated and formed asci with eight spores; however, the mating frequency varied greatly (Figure 3A). For example, cells mated most efficiently in the NBRC1713 strain (85.6% ± 6.8%; Figure 3A), whereas the FY33672, NBRC1646, and NBRC10527* strains were completely sterile (0%). Six of the eight strains isolated from *Drosophila* mated at a relatively high frequency (ranging from 14.4% ± 3.8% to 55.0% ± 7.7%), but the remaining two strains, TN40 and TN82, hardly mated on EMM2−N agar medium (Figure 3A).

We noticed that the strains isolated from *Drosophila* mated and sporulated even on YEA medium containing an abundant nitrogen source (Figure 3B); therefore, we also examined mating efficiency on YEA medium in the same way. Notably, strains TN33, TN83, TN84, TN104, TN108, and TN313 showed markedly high mating frequency under this condition (Figure 3C). In particular, approximately half of the cells of the TN33 strain mated on YEA medium after incubation at 30 °C for 48 hours (48.3% ± 6.3%). This tendency was also seen in FY32181*, the type strain of *S. japonicus* var. *versatilis* (Figure 3C). Interestingly, these strains that showed high mating frequency on the YEA medium also formed spores at high frequency after mating (Figure 3D). As far as we know, the mating response is not induced in laboratory strains of other fission yeast species under this condition. Thus, these data, showing that most of the strains isolated from *Drosophila* can sporulate even on YEA medium, may reflect a specific environment in *Drosophila*.

### 3.5. The TN33 Strain of S. japonicus var. japonicus Shows Iodine-Positivity

Sporulated colonies of *S. pombe* and *S. octosporus* are stained by iodine vapors [28] because the spores consist of a starch-like compound. Spores of the type strain of *S. japonicus* var. *versatilis* are also stained with iodine vapor; however, those of *S. japonicus* var. *japonicus* are not [15,16], although the underlying reason is unknown.

**Figure 4:**
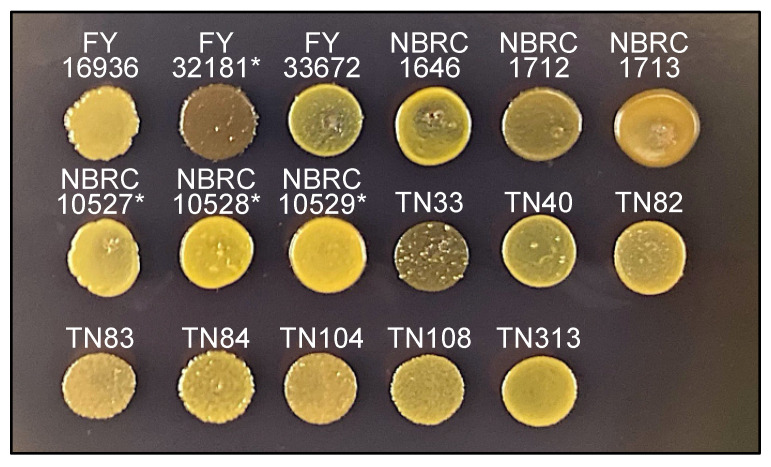
Iodine staining. Sporulating cells of 17 *S. japonicus* strains incubated on MEA medium at 30 °C for 2 days were stained by iodine vapor for 1 min. As reported previously, the type stain (FY16936) of *S. japonicus* var. *japonicus* was negatively stained, whereas the type stain (FY32181*) of *S. japonicus* var. *versatilis* was positively stained. Under this condition, TN33 was strongly positive; the others stains were negative (or weakly positive).

Next, therefore, we examined whether the 17 *S. japonicus* wild strains are stained by iodine vapors. As expected, after treatment of the strains on sporulation medium (MEA) with iodine vapor, colonies of the FY32181* strain were clearly stained, but those of the FY16936 strain were not (Figure 4). Unexpectedly, among the strains that were isolated from *Drosophila*, the TN33 strain was strongly stained dark brown by iodine vapor after 1 minute (Figure 4). Possibly, *S. japonicus* cells might mate repeatedly with each other in *Drosophila*, leading to the occurrence of a strain such as TN33 with a genetic background showing positive iodine staining.

### 3.6. Differences in Pheromone-Related Genes among S. japonicus Strains

The *S. japonicus* strains that we investigated showed diverse phenotypes in sexual reproduction (Figure 2, Figure 3 and Figure 4); therefore, we surmised that the mating pheromones and their corresponding receptor genes were diversified among the strains. Fission yeast has two mating types, Plus and Minus, which each secrete the respective mating pheromones *p*-factor and M-factor for mating [30,31]. Because the M-factor gene has not been identified in *S. japonicus*, we sequenced the P-factor gene (*map2^+^*), M-factor receptor gene (*map3^+^*), and P-factor receptor gene (*mam2^+^*) of all 17 strains and compared the nucleotide sequences with those of the FY16936 strain. Many nucleotide differences in the three genes were found among the strains (Table S2). As summarized in Table 2, the amino acid sequences of Map3 and Mam2 were relatively diversified; for example, the *map3* gene was identical in all four strains of *S. japonicus* var. *versatilis*, whereas there were many nucleotide differences and 14 amino acid differences between the Map3 proteins of *S. japonicus* var. *japonicus* and *versatilis* (Table S2). In addition, some mutations in the *mam2* genes were common to strains isolated in certain regions and countries (Table S2).

**Figure 5:**
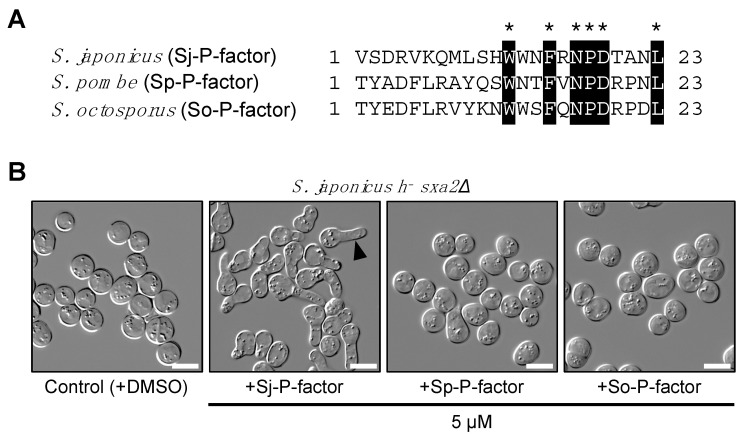
Shmooing assay using synthetic P-factor peptides. (**A**) Amino acid alignment of P-factor peptide from three *Schizosaccharomyces* species. Numerals to the right of each sequence indicate the length of the peptide product. Identical amino acids in the three peptides are highlighted by asterisks (*). (**B**) Shmoo formation in *S. japonicus* M cells lacking the *sxa2^+^* gene (*h^−^ sxa2Δ*). Cells were treated with each of the synthetic P-factors at 5 µM and incubated in EMM2*−*N medium with gentle shaking for 24 hours. Arrow indicates a representative shmooing cell. Scale bar, 10 μm.

In contrast, sequencing indicated that the *map2* gene of all 17 strains produced four copies of an identical mature P-factor peptide, VSDRVKQMLSHWWNFRNPDTANL (Figure 5A), although some missense and silent mutations were found in the pro sequences and mature pheromone sequences (Table S2). Therefore, we chemically synthesized this simple peptide and used it to treat *S. japonicus* cells. In *S. pombe*, P-factor is degraded outside the cell by the carboxyl peptidase Sxa2 [32,33,34]. Here, therefore, we constructed a heterothallic M-type strain (TS16) lacking the *sxa2^+^* gene (SJAG_00975) and examined whether the corresponding P-factor peptide of the three fission yeast species can be recognized by Mam2 of *S. japonicus* in the absence of Sxa2. The TS16 strain was treated with synthetic P-factors at 5 µM in EMM2−N medium and the formation of a shmoo – the mating projection that is elongated when yeast cells sense pheromones – was monitored. When treated with Sj-P-factor, *S. japonicus* cells showed sufficient shmoo formation, whereas the other two P-factor peptides, Sp-P-factor and So-P-factor, were not recognized at all (Figure 5B). These data indicate that Mam2 of *S. japonicus* specifically recognizes Sj-P-factor.

### 3.7. Diversify of S. japonicus Wild Strains in Nature

**Figure 6:**
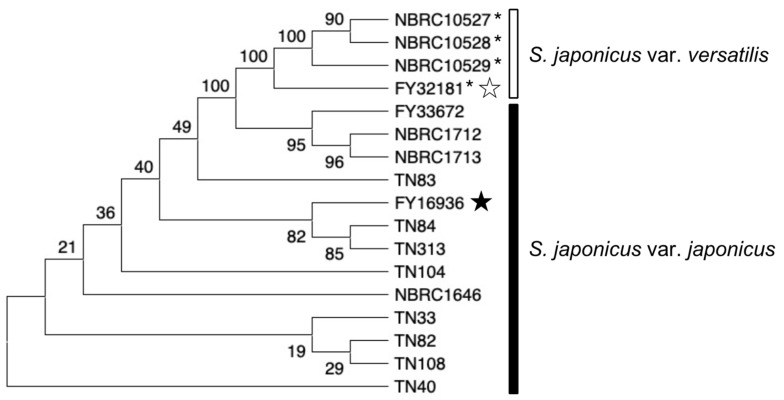
Evolutionary relationship of *S. japonicus* strains. Phylogenetic tree of the 17 *S. japonicus* wild strains investigated in this study. The analysis combined multiple sequence alignments for the *map2*, *map3*, and *mam2* genes. The evolutionary history was inferred by using neighbor-joining [35] and the Kimura 2-parameter method [36], and the number of base substitutions per site is shown. All ambiguous positions were removed for each sequence pair. In total, there were 2766 positions in the final data set. Evolutionary analyses were conducted in MEGA X [37,38]. Black and white stars indicate the type strain of *S. japonicus* var. *japonicus* and *S. japonicus* var. *versatilis*, respectively. *S. japonicus* var. *versatilis* strains were marked with an asterisk (*).

We constructed a phylogenetic tree based on the sequences of three genes (*map2*, *map3*, and *mam2*) in the 17 *S. japonicus* strains (Figure 6). In the tree, strains of each subspecies were clustered, while the evolutionary distance among the strains was not necessarily consistent with sampling locations. For example, the TN84 strain (isolated at RIKEN) is evolutionarily closer to the TN313 strain (isolated at Osaka University) than to the TN40 strain (isolated at RIKEN). These data suggest that *Drosophila* consume yeast while flying around the neighborhood, and accumulate many strains of yeast in their crop for a while. A strategy for isolating yeasts from highly mobile flies might be effective for the collection of yeasts showing various phenotypes.

## 4. Discussion

Yeasts are widely dispersed in nature across a variety of habitats and are commonly found on fruits and plants, in alcohol, as well as on the surface of the skin of animals and humans. In this study, we isolated eight strains from fruit flies sampled at two distinct locations in Japan, indicating that the fission yeast *S. japonicus* species may live symbiotically (or temporally) in *Drosophila*. As far as we know, this is the first report of the isolation of this species from insects.

It is well documented that *Drosophila* species in the wild use yeast as a food source [39,40,41]. Laboratory studies on the budding yeast *Saccharomyces cerevisiae* and *Drosophila melanogaster* have demonstrated that almost all vegetative cells are killed by passage through the gut in *D. melanogaster*, but spores show enhanced survival [24,42]. Spores are quiescent cells that have resistance to various environmental stimuli, probably due to their two-layer outer wall, which comprises chitosan [43] and a di-tyrosine (in *S. cerevisiae* [44]) or isp3 (in *S. pombe* [45]) polymer. Accordingly, spores have resistance to stresses such as ethanol, low and high pH, and high temperatures (>50 °C) [45,46,47]. These experimental data might explain our finding that *S. japonicus* strains isolated from *Drosophila* show elevated sporulation efficiency (Figure 3). Our sequencing analysis further showed that the strains highlighted from *Drosophila* have different genetic backgrounds (Table 2 and Table S2). Interestingly, a previous study reported that outbreeding of *S. cerevisiae* can be enhanced by the degradation of interspore bridges by *Drosophila* [24]. Therefore, we speculate that sporulation followed by mating is facilitated in *Drosophila*, where crossing between closely related species may frequently occur.

In many immobile organisms such as plants and fungi, the sexual reproduction phase (pollen and spores) is the mobile phase of the life cycle. A strategy to migrate to a new environment via predation by flies (or other animals and humans) is probably convenient because fries give yeast not only the ability to disperse but also a higher chance of migrating to a viable environment (e.g., the host’s preferred food) rather than just dispersing. Insects can increase the probability of outbreeding by flying from flower to flower in search of nectar, collecting yeasts and their spores, and then dropping them all in one place.

Isolation of yeasts from insects such as *Drosophila* will be beneficial for the collection of yeasts that have various phenotypes due to genetic diversification, such as the iodine-positive *S. japonicus* var. *japonicus* strain TN33 identified herein (Figure 4). Further detailed analysis of other phenotypes of these strains, such as nutritional requirement, mating type, and the effects of mutations in pheromone-related genes, should be conducted in the future. We hope that the information and resources presented in this study will provide a basis for future studies on the signal transduction mechanisms of sporulation, as well as the ecology and evolution of yeast.

## Figures and Tables

**Table 1:** *S. japonicus* wild strains used in this study.

Strain	Variety	Origin	Source	Reference
FY16936	*S. japonicus* var. *japonicus*	Japan, Kyushu University	strawberry	NBRP
FY32181 *	*S. japonicus* var. *versatilis*	USA	home-canned grape juice	NBRP
FY33672	*S. japonicus* var. *japonicus*	Japan, Suzuka College		NBRP
NBRC1646	*S. japonicus* var. *japonicus*	Japan	slime flux of tree	NBRC
NBRC1712	*S. japonicus* var. *japonicus*	Unknown		NBRC
NBRC1713	*S. japonicus* var. *japonicus*	Unknown		NBRC
NBRC10527 *	*S. japonicus* var. *versatilis*	Unknown	grape juice	NBRC
NBRC10528 *	*S. japonicus* var. *versatilis*	Portugal	white wine	NBRC
NBRC10529 *	*S. japonicus* var. *versatilis*	Unknown	slime flux, Ulmus carpinifolia	NBRC
TN33	*S. japonicus* var. *japonicus*	Japan, RIKEN	*Drosophila*	This study
TN40	*S. japonicus* var. *japonicus*	Japan, RIKEN	*Drosophila*	This study
TN82	*S. japonicus* var. *japonicus*	Japan, RIKEN	*Drosophila*	This study
TN83	*S. japonicus* var. *japonicus*	Japan, RIKEN	*Drosophila*	This study
TN84	*S. japonicus* var. *japonicus*	Japan, RIKEN	*Drosophila*	This study
TN104	*S. japonicus* var. *japonicus*	Japan, RIKEN	*Drosophila*	This study
TN108	*S. japonicus* var. *japonicus*	Japan, RIKEN	*Drosophila*	This study
TN313	*S. japonicus* var. *japonicus*	Japan, Osaka University	*Drosophila*	This study

In this study, to distinguish between the two subspecies more easily, *S. japonicus* var. *versatilis* strains were marked with an asterisk (*).

**Table 2:** Polymorphisms of pheromone-associated genes in the 17 *S. japonicus* strains investigated.

Gene	Amino acid Substitution	No. of Strains	Strain
*map2* (SJAG_00781)	WT	15	FY16936, FY32181 *, FY33672, NBRC1646, NBRC1712, NBRC1713, NBRC10527 *, NBRC10529 *, TN33, TN40, TN82, TN83, TN84, TN104, TN108
	S22N/N32V	1	NBRC10528 *
	A29S	1	TN313
*map3* (SJAG_01345)	WT	11	FY16936, FY33672, NBRC1646, NBRC1713, TN40, TN82, TN83, TN84, TN104, TN108, TN313
	S3P/V4E/M27L/V43L/I54V/I59V/F117L/I121V/I125V/I179V/T222S/M223L/M261L	4	FY32181 *, NBRC10527 *, NBRC10528 *, NBRC10529 *
	A44S/L169V	1	NBRC1712
	I328V	1	TN33
*mam2* (SJAG_01928)	WT	10	FY16936, FY32181 *, TN33, TN40, TN82, TN83, TN84, TN104, TN108, TN313
	A13T/T28V/V170I	1	FY33672
	A185G	1	NBRC1646
	V170I	1	NBRC1712
	A13T/V170I	2	NBRC1713, NBRC10527 *
	A13T/V170I/R319G	1	NBRC10528 *
	A13T/V170I/S277W/L283F R319G/S330F	1	NBRC10529 *

*S. japonicus* var. *versatilis* strains were marked with an asterisk (*).

## Data Availability

All relevant data are included in the paper and its Supplementary Materials.

## Supplementary Materials

The following are available online at https://www.mdpi.com/article/10.3390/jof7050350/s1, Table S1: Primers used in this study. Table S2: Nucleotide sequences of three pheromone-associated genes (*map2*, *map3*, and *mam3*) in 17 wild *S*. *japonicus* strains, Table S3: Numerical data for all of the graphs presented in this study.
